# A Synergistic Examination of Excitatory‐Inhibitory Balance in First‐Episode Schizophrenia Merging 7‐Tesla Magnetic Resonance Spectroscopic Imaging and Magnetoencephalography

**DOI:** 10.1002/brb3.70735

**Published:** 2025-08-04

**Authors:** Alfredo L. Sklar, Annie Blazer, Warren Snover, Fran López‐Caballero, Mark Curtis, Brian A. Coffman, Hoby H. Hetherington, Chan‐Hong Moon, Dean F. Salisbury, Deepak K. Sarpal

**Affiliations:** ^1^ Department of Psychiatry Pittsburgh University of Pittsburgh School of Medicine Pittsburgh Pennsylvania USA; ^2^ Department of Psychological and Brain Sciences Washington University St. Louis Missouri USA; ^3^ Resonance Research Incorporated Billerica Massachusetts USA; ^4^ Department of Radiology University of Missouri Columbia Missouri USA; ^5^ Department of Radiology Pittsburgh University of Pittsburgh School of Medicine Pittsburgh Pennsylvania USA

**Keywords:** 7‐Tesla magnetic resonance spectroscopic imaging, evoked gamma‐band response, first‐episode schizophrenia, gamma‐aminobutyric acid, glutamate, magnetoencephalography

## Abstract

**Introduction:**

Excitatory/inhibitory (E/I) imbalance is a proposed neural disruption in schizophrenia supported by magnetic resonance spectroscopic imaging (MRSI) evidence of altered gamma‐aminobutyric acid (GABA) and glutamate (Glu) levels. However, there exists a paucity of data linking these abnormalities to impaired in vivo brain function putatively reflecting E/I imbalance. Here, associations between GABA/Glu and the evoked early auditory gamma‐band response (EAGBR) were examined in first‐episode schizophrenia (FESz).

**Methods:**

Twelve FESz underwent resting‐state 7T‐MRSI and magnetoencephalography (MEG) recorded during an auditory oddball task. MRSI spectra and source‐localized MEG data were extracted from overlapping regions of left (LH) and right (RH) superior temporal sulcus. Associations between evoked EAGBR power and GABA/Glu, GABA/Cre, and Glu/Cre ratios were assessed. The Brief Psychiatric Rating Scale (BPRS) and Global Functioning: Role/Social (GF: Role/Social) scales were collected.

**Results:**

GABA/Glu ratios were inversely correlated with EAGBR power (LH: *ρ* = −0.87; RH: *ρ* = −0.85). LH GABA/Glu ratios were also inversely correlated with BPRS scores (*ρ* = −0.64) and positively correlated with GF: Social (*ρ* = 0.64) scores while RH EAGBR power was positively correlated with BPRS scores (*ρ* = 0.70). Stepwise linear regressions suggest these relationships were driven primarily by GABA concentrations.

**Conclusion:**

Associations in FESz between GABA/Glu ratios, clinical ratings, and EAGBR power suggests this evoked gamma‐band response reflects cortical hyperexcitability within the auditory system that is closely tied to disease debility at this early illness stage. These data highlight the potential of merging high‐precision, in vivo neuro‐chemical assays via 7‐Tesla MRSI and physiological measures from MEG to validate established cellular models of disease.

## Graphical abstract

A merged analysis of 7T MRSI and MEG recordings revealed an association between smaller GABA/Glutamate ratios and the evoked early auditory gamma‐band response (EAGBR), a potential marker of cortical hyperexcitability, in first‐episode schizophrenia. This relationship, and associated clinical outcomes, appear to be driven by GABA levels.

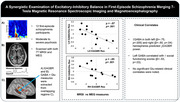

## 1 Introduction

Imbalanced excitatory and inhibitory (E/I) neurotransmission has long been considered a neuropathological hallmark of schizophrenia (Olney and Farber 1995). This theory is grounded in work identifying NMDA receptor hypofunction, a deficit resulting in impaired inhibitory interneuron transmission and disinhibition of pyramidal cells leading to downstream hyperexcitability of neural circuits (Cohen et al. 2015; Gonzalez‐Burgos and Lewis 2012). Ample evidence from human magnetic resonance spectroscopy demonstrating elevated glutamate levels in first‐episode schizophrenia (FESz) has provided important in vivo support for E/I imbalance in the disorder (Théberge et al. 2002; Tandon et al. 2013; de la Fuente‐Sandoval et al. 2013; Chen et al. 2023).

Quantification of gamma‐aminobutyric acid (GABA), however, remains a challenge at lower field strengths (1.5–4T) due to contamination with macromolecules and lower spatial resolution requiring large, singular regions of interest (ROIs). Findings from high‐field, 7‐Tesla magnetic resonance spectroscopic imaging (MRSI) demonstrate acquisition of multivoxel measures of GABA with higher accuracy and smaller ROIs, with preliminary evidence for reduced GABA concentrations in FESz (Mayeli et al. 2022; Sonnenschein et al. 2022; A. Wang et al. 2019). Both GABA and Glu levels also fluctuate longitudinally over early illness course and may provide insights into the impact of treatment outcomes and/or disease chronicity (Merritt et al. 2021; Egerton et al. 2017). However, measures of neural processing sensitive to E/I balance with a spatial resolution compatible to MRSI are needed to provide a critical link between neurochemical disruptions and disease burden.

Local synchronization of gamma‐band oscillations represents a potential candidate marker for balanced E/I neurotransmission that facilitates efficient information transfer between neuronal ensembles. This synchronized neural oscillation is dependent on coordination between glutamatergic pyramidal cells and GABAergic interneurons, with the latter imposing rhythmic inhibition on the excitatory drive of the former to frame the gamma cycle (Bartos et al. 2007; Fries et al. 2007). In schizophrenia, stimulus‐induced gamma‐band responses (stimulus‐locked, but not phase‐dependent across trials) typically recorded using EEG exhibit impairment across illness stage (Minzenberg et al. 2010; Cho et al. 2006). This response is believed to reflect binding of stimulus features to generate a cohesive perceptual experience and has been associated with cognitive deficits of the disorder (Senkowski and Gallinat 2015). However, while previous reports have identified an association between induced gamma‐band power and GABA levels (Balz et al. 2016; Muthukumaraswamy et al. 2009), few studies have documented this relationship in schizophrenia.

Unlike induced activity, the evoked gamma response is closely linked to the timing and characteristics of stimulus presentation. The contribution of sensory‐level processing deficits to downstream disease symptoms and functional impairments (Hamilton et al. 2018; Sklar et al. 2023) and their potential to elucidate fundamental circuit dysfunctions in schizophrenia (Javitt and Freedman 2015) has become increasingly clear. Impairment of the auditory steady‐state response (ASSR), a gamma oscillation within auditory cortex entrained to the presentation frequency of a stimulus, has been observed across illness stage (Onitsuka et al. 2022). In contrast, the early auditory gamma‐band response (EAGBR), a bottom‐up measure of local gamma‐burst activity (∼50 ms poststimulus) evoked by discrete stimuli, may be sensitive to illness chronicity. While deficits have been observed in early course schizophrenia (Leicht et al. 2010; Roach and Mathalon 2008), the EAGBR appears to be preserved proximal to disease onset (Gallinat et al. 2004; Spencer et al. 2008; Sklar et al. 2024b) with evidence of longitudinal decline over the first year of illness (Oribe et al. 2019; Sklar et al. 2024a). Interestingly, associations between larger EAGBRs and worse clinical outcomes suggest this stimulus‐evoked response may provide a marker of pathologic cortical hyperexcitability (Sklar et al. 2024a, 2024b; Taylor et al. 2013). These findings are consistent with observations of excessive glutamatergic transmission that moderates over time with response to treatment (Merritt et al. 2019). Unfortunately, while a positive correlation between glutamate levels and evoked gamma‐band power has been reported in healthy adults (Lally et al. 2014), this relationship has not been explored in schizophrenia.

In this preliminary analysis, we utilized 7T‐MRSI and magnetoencephalography (MEG) recordings from overlapping ROIs to examine the neurophysiological concomitants of in vivo, neurochemical E/I imbalance in FESz. We hypothesized that cortical hyperexcitability, indexed by lower GABA/glutamate ratios would be associated with larger EAGBRs co‐localized within the auditory system as well as more severe illness‐related debility in FESz. The complexity of the disease and the organ in question demands utilization of multimodal methodologies which, on their own, can offer only limited insights into the nature of neuropsychopathology. While MEG and 7T MRSI pose logistical and technological challenges limiting their broader utilization, merging these techniques leverages their superb anatomic fidelity and offers a novel approach to isolate disruptions of E/I balance, its consequences for brain function, and its contribution to disease burden in schizophrenia.

## 2 Methods

### 2.1 Participants

Twelve FESz were included. Participants were enrolled in two separate studies and were included in the current analysis if they completed both MEG and 7T‐MRSI scan protocols as part of each. Participants were between the ages of 18 and 35, carried a DSM IV diagnosis of either schizophrenia, schizophreniform disorder, or schizoaffective disorder; cumulative lifetime antipsychotic medication exposure of ≥ 1 year; and presented with moderate to severe psychosis defined by a score of at least 4 on one or more psychosis relevant Brief Psychiatric Rating Scale (BPRS) items (Woerner et al. 1988). Participants were excluded for a history of concussion with sequelae, history of substance dependence, a positive urine drug screen on day of testing aside from cannabis, < 9 years of education, or abnormal hearing as assessed by audiometry.

Clinical assessments included the Structured Clinical Interview for DSM‐IV (SCID‐IV), BPRS, Global Functioning: Role and Social (GF: Role/Social) scales, and the MATRICS Consensus Cognitive Battery (MCCB). Diagnoses were confirmed via consensus by expert clinicians and the SCID‐IV.

This study was approved by the University of Pittsburgh Institutional Review Board, and all participants provided written informed consent prior to completing study procedures.

### 2.2 Auditory Task

An auditory oddball task consisting of 340 standard (1000 Hz, 50 ms duration, 10 ms rise/fall) and 60 deviant (1200 Hz, 50 ms duration, 10 ms rise/fall) tones with a variable stimulus onset asynchrony of 1050–1550 ms was used to elicit the EAGBR. Participants were instructed to respond to deviant tones via button‐press.

### 2.3 MEG Data Acquisition and Source Localization

MEG acquisition and processing protocols were identical to our previous investigation (Sklar et al. 2024b) apart from the region used to evaluate the EAGBR. MEG was recorded using an Elekta‐Neuromag Vectorview system. Bipolar leads were placed at the outer canthi of both eyes and single channel leads were placed below the left eye and on the left clavicle. Four head positioning indicator (HPI) coils were placed on the scalp and a 3D digitizer was used to record their location. Preprocessing included Neuromag MaxFilter software (http://imaging.mrc‐cbu.cam.ac.uk/meg/Maxfilter_V2.2) to correct for head motion and use of an adaptive mixture independent component analysis to isolate and remove artifacts. Further sensor‐level processing, conducted in Brainstorm (Tadel et al. 2011), included application of low‐pass (100 Hz) and notch (60 Hz) filters, segmentation of standard tone trial data (−300 to 600 ms), baseline‐correction (−150 to 0 ms), and rejection of trials exceeding ± 5pT.

**FIGURE 1:**
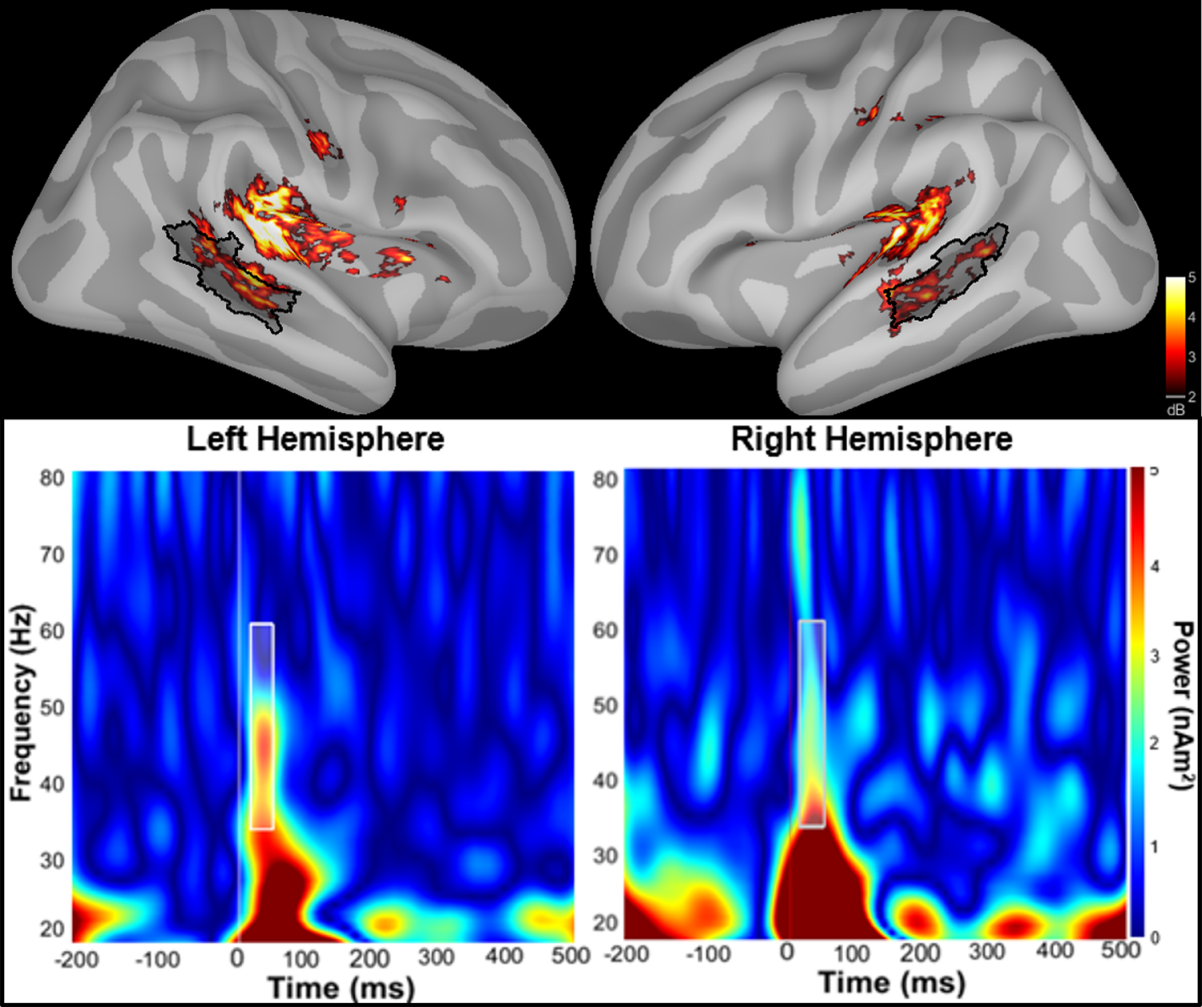
(Top) The early auditory gamma‐band response (EAGBR) localized on the cortical surface. The superior temporal sulcus (STS) parcel used for merged MEG and 7T‐MRSI data is shaded in black. (Bottom) Spectrograms extracted from the left and right hemisphere STS parcels. The white box indicates the frequency and time boundaries used to define the EAGBR.

MRI scans were obtained to localize cortical sources of MEG sensor data using a Siemens Magnetom Prisma Fit 3T system. Data were processed according to Human Connectome Project (HCP) pipelines (Glasser et al. 2013) to obtain HCP cortical parcellations for each participant. Scans included T1‐ and T2‐weighted images and a 10 min resting‐state fMRI scan. MEG sensor data was registered to each participant's MRI scan. A forward solution was modeled as overlapping spheres based on sensor locations and a noise covariance matrix was calculated from baseline. Source were localized using the minimum norm estimation with an orientation constraint of 0.2. Regions of auditory association cortex including STS_dorsal_posterior and STS_ventral_posterior HCP regions were merged to create our STS parcel of interest (Figure 1). While early auditory cortices within the superior temporal gyrus typically elicit a larger EAGBR, this region of the STS was selected given its overlap with ROIs used to obtain MRSI data (verified using center of mass MNI coordinates) and an identifiable EAGBR signal (Figure 1).

A Morlet wavelet transform (*f* = 1 Hz; FWHM = 3 sec) was applied at 1 Hz steps from 20 to 80 Hz to source‐localized data. EAGBR power, extracted from the trial‐averaged sources, was calculated at each cortical vertex, averaged across the STS parcel, and baseline (−150 to 0 ms) corrected. The EAGBR was defined using a time (20–60 ms) and frequency (35–60 Hz) windows (Figure 1) based on previous analyses that included the current study sample (Sklar et al. 2024a, 2024b).

### 2.4 7‐T MRSI Acquisition and Processing

Full description of 7T‐MRSI methods have been previously published (Mayeli et al. 2022; Sonnenschein et al. 2022; Parr et al. 2024; Perica et al. 2022; McKeon et al. 2024; Quiñones et al. 2021). Briefly, a Siemens 7‐Tesla Magnetom whole body human scanner was utilized to acquire data with an 8‐channel parallel transmit (pTx) system and an 8 × 2 transceiver array (two rows with eight coils/row, Resonance Research Inc.). Higher degree/order B0 shimming included first–fourth‐order shims and partial fifth‐order shims with a very high order shimming insert (Resonance Research Inc.; 41). Extra‐cranial tissue suppression was achieved using B1 shimming with two different B1^+^ distributions: a uniform B1 distribution was used to excite the brain, while a ring‐shaped B1 distribution was used to selectively suppress tissues outside the brain (Hetherington et al. 2010). Water suppression was achieved using a broad‐band semi‐selective refocusing pulse as well as a frequency‐selective inversion pulse (Hetherington et al. 2010). MRSI data was collected from a 10 mm thick slab along the thalamic plane with a double‐echo J‐refocused coherence transfer sequence (14 min and 30 s long, TR of 1500 ms, TE of 34 ms, matrix size = 24 × 24, field of view of 216 × 216 mm^2^) (Pan et al. 2010). This sequence was designed to optimize detection of metabolites across the spectrum, over traditional methods (e.g. MEGA_PRESS) given B0 field inhomogeneities typical of 7T MRSI planes. An MP2RAGE sequence (Marques et al. 2010) (TR 6000 ms; TI 800 and 2700 ms) with 1.0 mm isotropic resolution in an FOV of 240 × 240 × 192 mm was used for anatomical identification.

Spectral data for GABA and Glu in the ROIs were extracted using LCModel, per previous studies (Mekle et al. 2009; Provencher 2001). Spectral data included default macromolecule components and 14 basis metabolite functions (*N*‐acetylaspartate, *N*‐acetylaspartylglutamate, aspartate, lactate, creatine (Cre), GABA, glucose, Glu, glutamine, glutathione, glycerophosphorylcholine, phosphorylcholine, myoinositol, and taurine). Cramer‐Rao lower bounds (CRLB) values were obtained from LCModel indicate fit between the measurement of the metabolite and the ideal spectrum for that metabolite.

**FIGURE 2:**
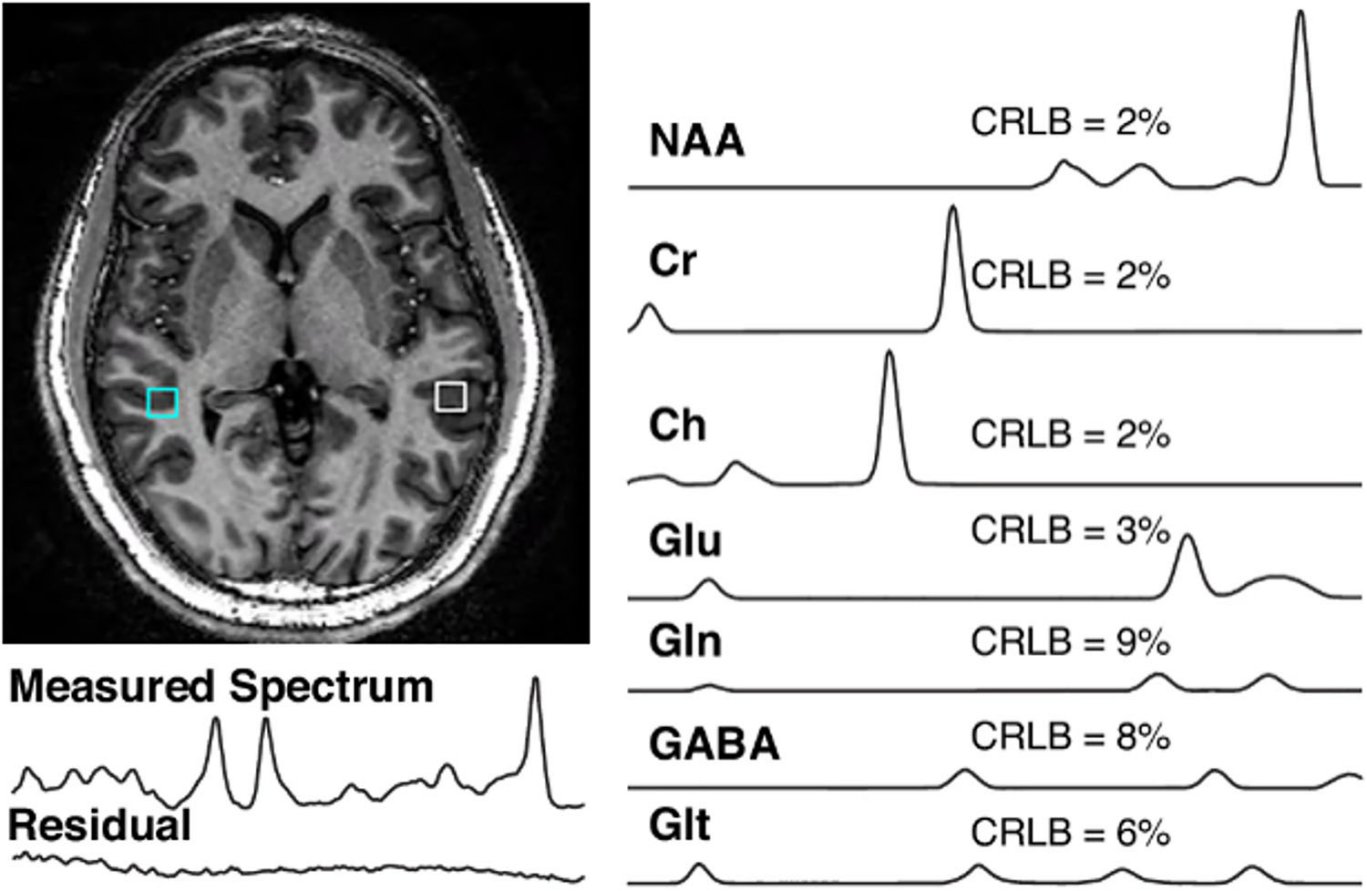
Example of typical voxels identified in the MRSI plane. The measured spectrum (left), and separate spectra for each metabolite extracted from a voxel (right) are displayed.

Bilateral MRSI ROIs (9 × 9 × 10 mm) were visually identified along the acquired slab in each participant's native space and centered in the temporal lobe in the superior temporal sulcus (Figure 2). For purposes of this study, spectra were quantified and Glu, GABA, and Cre values were extracted (Figure 2). Neurotransmitter levels are expressed as the metabolite relative to Cre (i.e., Glu/Cr and GABA/Cr), which allows for shorter acquisition time in comparison to using a water reference, and controls for inter‐subject variability. We chose Cre as the reference due to its strong signal and reliable chemical shift (Mayeli et al. 2022; Sonnenschein et al. 2022; Parr et al. 2024; Perica et al. 2022; McKeon et al. 2024; Quiñones et al. 2021). Metabolites with a CRLB value of less than 20 were included, consistent with previous literature (Kumar et al. 2020; Wijtenburg et al. 2021). Finally, anatomical locations, identified in native space, were warped to MNI space with Advanced Normalization Tools (ANTs) to co‐localize with MEG data (Avants et al. 2011).

### 2.5 Data Analysis

Demographic and clinical variables are presented in Table 1. Our primary analysis consisted of Spearman rank‐order partial correlations between left and right hemisphere (LH/RH) GABA/Glu ratios, EAGBR power, and clinical assessments controlling for percent gray matter within each voxel. FDR correction was used to correct for multiple comparisons with a *q* < 0.1 considered significant. Full correlation results are depicted in Table 2. Secondary analyses were conducted utilizing a stepwise multiple linear regression to examine individual contributions of GABA and Glu, normalized using Cre levels, to each significant relationship. Regression analyses are summarized in Table 3.

**TABLE 1:** Demographic and clinical characteristics.

Female/male	4/8
Education (years)	12.64 ± 1.8
Age (years)	22.55 ± 5.3
MCCB‐total	25.50 ± 14.0
GF: Role	4.25 ± 1.7
GF: Social	4.42 ± 2.0
Race/ethnicity (W/B/A/H/N/I/U)	4/4/2/0/0/1/1
BPRS total	49.08 ± 8.0
Medicated	11/12
CPZ (mg/day)	183.09 ± 128.9
DUP (months)	13.22 ± 10.1

Abbreviations: BPRS, Brief Psychiatric Rating Scale; CPZ, chlorpromazine equivalents; DUP, duration of untreated psychosis; GF: Role, Global Functioning: Role Scale; GF: Social, Global Functioning: Social Scale; MCCB, MATRICS Consensus Cognitive Battery.

**TABLE 2:** Partial correlations between GABA/Glu ratio measures, the EAGBR and clinical ratings.

	EAGBR	BPRS	GF:R	GF:S	MCCB
GABA/Glu					
Left	***ρ* = −0.87** ^*^	***ρ* = −0.64** ^*^	***ρ* = 0.62**	***ρ* = 0.64** ^*^	*ρ* = 0.53
Right	***ρ* = −0.85** ^*^	*ρ* = −0.28	*ρ* = −0.08	*ρ* = 0.00	*ρ* = 0.11
EAGBR					
Left	—	*ρ* = 0.57	*ρ* = −0.32	*ρ* = −0.51	*ρ* = −0.16
Right	—	***ρ* = 0.70** ^*^	*ρ* = −0.16	*ρ* = −0.41	*ρ* = −0.19

*Note*: **Boldface**: uncorrected *p* < 0.05.

Abbreviations: BPRS, Brief Psychiatric Rating Scale; EAGBR, early auditory gamma‐band response; GF:R, Global Functioning: Role Scale; GF:S, Global Functioning: Social Scale; MCCB, MATRICS Consensus Cognitive Battery.

* survived FDR correction.

**TABLE 3:** Stepwise regression statistics for prediction of EAGBR and clinical ratings.

	Independent variable	Adjusted *R* ^2^	Standardized *β*
LH EAGBR	LH GABA/Cre	0.51^*^	−0.75^*^
	LH Glu/Cre		−0.24
RH EAGBR	RH GABA/Cre	0.30^*^	−0.60^*^
	RH Glu/Cre		0.35
BPRS	LH GABA/Cre		
	LH Glu/Cre		
GF: Social	LH GABA/Cre	0.33^*^	0.63^*^
	LH Glu/Cre		−0.14

Abbreviations: BPRS, Brief Psychiatric Rating Scale; EAGBR, early auditory gamma‐band response; GF: Social, Global Functioning: Social Scale.

* *p* < 0.05.

## 3 Results

### 3.1 Correlations With GABA/Glu Ratio

**FIGURE 3:**
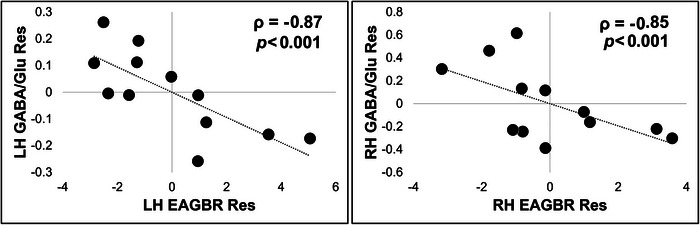
Partial correlations between left and right hemisphere GABA/glutamate ratios and the EAGBR controlling for percent gray matter.

GABA/Glu ratios in both LH (*ρ* = −0.87, *p* < 0.001, 95% CI: −0.97 to −0.50) and RH (*ρ* = −0.85, *p* < 0.001, 95% CI: −0.96 to −0.45) STS were inversely correlated with EAGBR power (Figure 3). LH GABA/Glu ratios were inversely correlated with BPRS (*ρ* = −0.64, *p* = 0.03, 95% CI: −0.90 to −0.04) and positively correlated with GF: Social (*ρ* = 0.64, *p* = 0.03, 95% CI: 0.04–0.90) scores. RH EAGBR power was positively correlated with BPRS scores (*ρ* = 0.70, *p* = 0.01, 95% CI: 0.14–0.92).

### 3.2 Correlations With Normalized GABA and Glu Levels

GABA/Cre ratios in both LH (*β* = −0.75, *p* = 0.005) and RH (*β* = −0.60, *p* = 0.04) predicted weaker EAGBR power. LH GABA/Cre also predicted improved GF: Social scores (*β* = −0.63, *p* = 0.03). Glu/Cre ratios did not serve as significant predictors of EAGBR and neither RH GABA/Cre nor Glu/Cre predicted GF: Role scores.

## 4 Discussion

The present investigation merged 7T‐MRSI and MEG recordings, leveraging the exquisite spatiotemporal resolution of these measures to isolate a functional measure of E/I imbalance in FESz. Preliminary analyses revealed associations between the GABA/Glu ratio, clinical ratings, and EAGBR power suggesting this evoked gamma‐band response reflects cortical hyperexcitability within the auditory system that is closely tied to disease debility at this early illness stage. Findings from this unique dataset, merging direct, high signal‐to‐noise measures of neurophysiology and neurochemistry, provide valuable insights into the neural underpinnings of symptoms and functional decline in schizophrenia.

Despite its modest sample size, the current results suggest the EAGBR approximates E/I balance in FESz. This evoked sensory response is preserved (Gallinat et al. 2004; Sklar et al. 2024a, 2024b), and in certain cases elevated (Sklar et al. 2024), relative to healthy adults at disease onset and is associated with more severe illness (Sklar et al. 2024a, 2024b; Taylor et al. 2013). In light of these recent findings, the inverse correlations between GABA/Glu ratios and the EAGBR in addition to their associations with clinical outcomes in the present sample provide evidence that this neurophysiologic measure of auditory processing reflects pathologic cortical hyperexcitability at this early illness stage. Consistent with previous literature documenting decreased GABA levels in FESz (Chiu et al. 2018; M. Wang et al. 2023), observed relationships between GABA/Glu and the EAGBR were predominantly driven by reduced GABA indicating neural disinhibition. While elevated Glu levels have also been reported at and even prior to disease onset (Théberge et al. 2002; Tandon et al. 2013; Chen et al. 2023), RH Glu/Cre did not predict the RH EAGBR in the current sample.

Interestingly, recent evidence suggests the EAGBR diminishes over the first year of illness (Oribe et al. 2019; Sklar et al. 2024a) and this decrease may be associated with symptom reduction (Sklar et al. 2024b). While evidence for longitudinal progression of the GABA/Glu ratio in FESz is lacking and results for GABA levels remain equivocal (Bojesen et al. 2024; Sonnenschein et al. 2022; A. Wang et al. 2019), elevated Glu levels noted at disease onset have been shown to normalize over time (Théberge et al. 2002; de la Fuente‐Sandoval et al. 2013; Merritt et al. 2021). Regardless of whether this change represents a therapeutic response or natural disease progression, it might help explain the previously noted reduction of the EAGBR and associated symptoms over time.

Certain limitations arose due to attempts at merging unique datasets across study protocols. Unfortunately, examination of both 7T‐MRSI and MEG data within the same individuals resulted in a relatively small FESz sample and a lack of a control comparison control group. The small sample likely limited our ability to detect relationships between MRSI, MEG, and clinical measures as evidenced by wide CIs and fairly large correlations that did not achieve significance. Furthermore, the lack of a healthy control sample limits the interpretation and clinical relevance of our findings. Work is ongoing to enhance collaboration and data collection from participants across laboratories and modalities at our institution to address this limitation. In addition, the MRSI voxel, which was limited to the acquired plane across the brain, restricted our EAGBR analysis to a region of the STS despite the strongest responses typically being generated by primary auditory cortex. However, well‐defined EAGBRs were still observed within this region of the broader auditory network (Figure 1), providing valuable insights into the detailed neurochemical concomitants of primary sensory processes in FESz. Despite these limitations, the current findings emphasize the value and feasibility of merging high‐precision, multimodal datasets to the pursuit of novel discoveries in the study of schizophrenia.

## Author Contributions

**Alfredo L. Sklar**: writing – original draft, formal analysis, methodology, visualization, data curation. **Annie Blazer**: writing – review and editing, project administration, data curation. **Warren Snover**: project administration, writing – review and editing, data curation. **Fran López‐caballero**: writing – review and editing, project administration, data curation. **Mark Curtis**: writing – review and editing, project administration, data curation. **Brian A. Coffman**: writing – review and editing, supervision, software, methodology, project administration. **Hoby H. Hetherington**: writing—review and editing, methodology. **Chan‐Hong Moon**: writing—review and editing, methodology, data curation, project administration. **Dean F. Salisbury**: conceptualization, funding acquisition, writing—review and editing, supervision, project administration. **Deepak K. Sarpal**: conceptualization, funding acquisition, writing—review and editing, project administration, supervision.

## Peer Review

The peer review history for this article is available at https://publons.com/publon/10.1002/brb3.70735.

## Data Availability

The data that support the findings of this study are available from the corresponding author upon reasonable request.
